# Supplementary material for: Overcoming the IOTLB wall for multi-100-Gbps Linux-based networking
Source: PeerJ Comput Sci. 2023 May 16;9:e1385. doi: 10.7717/peerj-cs.1385 (PMC10280580; doi:10.7717/peerj-cs.1385)
Supplement: Supplemental Information 1 [file peerj-cs-09-1385-s001.pdf]

A TESTBED ILLUSTRATION AND ADDITIONAL RESULTS

Figure 17 illustrates our testbed. We use the main slot of a Socket Direct ConnectX-6 for 200-Gbps experiments and a normal ConnectX-6 NIC with custom Dell firmware for 100-Gbps experiments.

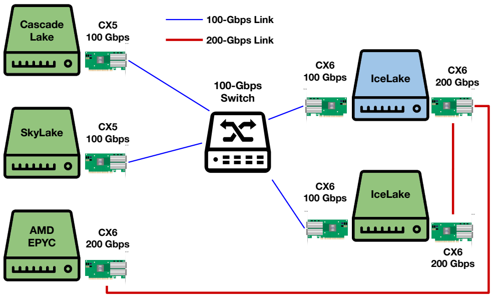

Figure 17. Our testbed. Green servers represent DUTs. CX5 and CX6 stand for ConnectX-5 and ConnectX-6, respectively.

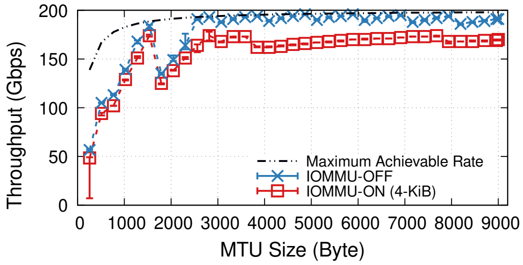

Figure 18. IOMMU imposes performance overheads at all MTU sizes, but with a larger absolute throughput drop for MTU sizes larger than ~3000 B.

Figure 18 shows the throughput for a fine-grained sweep of MTU values when the NIC uses 1024 RX descriptors per queue (*i.e.,* 32×1024 RX descriptors). Looking at the throughput values, we notice jumps at page splitting points. The `mlx5` driver splits pages based on the Hardware (H/W) MTU size that is 22 B larger than the value specified by the software (*e.g.,* via `ifconfig`). The extra 22 B ensures that the H/W buffer has enough space for the 14-B Ethernet header, 4-B VLAN header, and 4-B Ethernet Frame Check Sequence (FCS). The `mlx5` driver uses 512-B buffers for small H/W MTUs, but switches to 1024-B, 2048-B, and 4096-B buffers when the H/W MTU size exceeds 128, 640, and 1664 bytes. The driver uses a scatter-gather technique (*e.g.,* it uses multiple 256-B buffers) for "Jumbo" frames, *i.e.,* H/W MTU sizes >3712 B.
