# Supplementary material for: Overcoming the IOTLB wall for multi-100-Gbps Linux-based networking
Source: PeerJ Comput Sci. 2023 May 16;9:e1385. doi: 10.7717/peerj-cs.1385 (PMC10280580; doi:10.7717/peerj-cs.1385)
Supplement: Supplemental Information 2 [file peerj-cs-09-1385-s002.pdf]

B IOTLB OVERHEADS IN DPDK

For the sake of completeness, this section examines a kernel-bypass framework, Data Plane Development Kit (DPDK), to examine IOMMU in a high-performance & low-overhead setting. Additionally, using DPDK provides us with greater flexibility in memory management, enabling us to natively compare the impact of different (huge)page sizes (*i.e.,* 4-KiB & 2-MiB) on the performance of IOTLB. Furthermore, since DPDK allocates buffers contiguously (especially when we reserve hugepages at boot time), changing the (huge)page size will primarily show the impact of IOTLB misses. We use a DPDK-based packet-processing framework, FastClick Barbette et al. (2015), to generate & forward fix-sized packets at line rate. By doing so, we can benefit from all software optimizations shipped within FastClick and achieve the maximum achievable rate with DPDK Farshin et al. (2021).

Figure 19 shows the throughput and median latency of a server running an L2 forwarder application that receives fixed-size packets with 16 cores and 1024 RX descriptors per core, mirrors the MAC address of received packets and sends them back to the packet generator. These results show that kernel-bypass frameworks also experience similar performance degradations when they do not use hugepage IOTLB mappings. Note that the exact numbers may not match the iPerf results, as DPDK is less CPU-bound (*i.e.,* it achieves higher throughput with hugepages) and is less efficient without hugepages (*i.e.,* it achieves lower throughput in 4-KiB mode Yao and Hu (2018)).

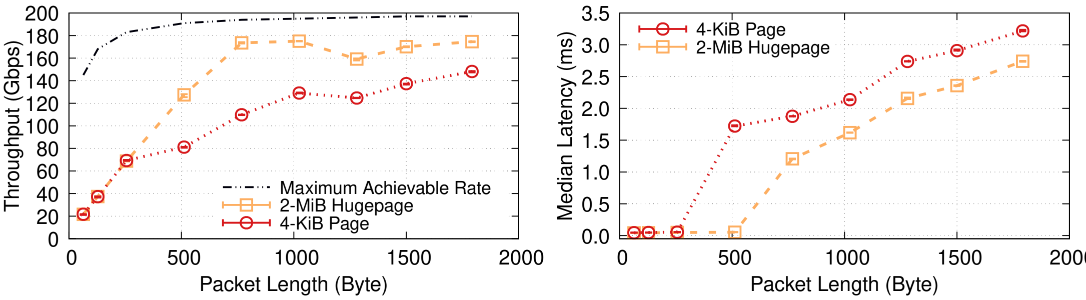

(a) Throughput. **(b)** Median latency.

Figure 19. DPDK also experiences performance degradation with IOMMU when it uses 4-KiB pages.
