# Supplementary material for: Overcoming the IOTLB wall for multi-100-Gbps Linux-based networking
Source: PeerJ Comput Sci. 2023 May 16;9:e1385. doi: 10.7717/peerj-cs.1385 (PMC10280580; doi:10.7717/peerj-cs.1385)
Supplement: Supplemental Information 3 [file peerj-cs-09-1385-s003.pdf]

C PAGE (DE)ALLOCATION VIA PAGE POOL API

Page Pool provides an API to device drivers to (de)allocate pages in an efficient, and preferably lockless way (see Figure 20 for details in Linux kernel v5.15); the driver then splits each page into single or multiple buffers based on a given MTU size, see Section 3.2. A page pool is composed of two main data structures: (i) a fixed-size lockless array/Last In, First Out (LIFO) (*aka* cache) and (ii) a variable-sized ring implemented via a `ptr_ring` data structure that is essentially a limited-size First in, First Out (FIFO) with spinlocks and that facilitates synchronization by using separate locks for consumers & producers. By default, the cache can contain up to 128 pages, and the size of the ring is determined based on the number of RX descriptors and MTU size. The purpose of the page pool is to (i) efficiently allocate pages from the cache without locking and (ii) use the ring to recycle returned pages. To avoid synchronization problems, each page pool should be connected to only *one* RX queue, thus it is protected by NAPI scheduling. The Page Pool API is mainly used to allocate memory at the granularity of a page; however, recent Linux kernels support page fragments that make it possible to allocate an arbitrary-sized memory chunk from an order-n page, *i.e.,* 2^n contiguous 4-KiB pages.

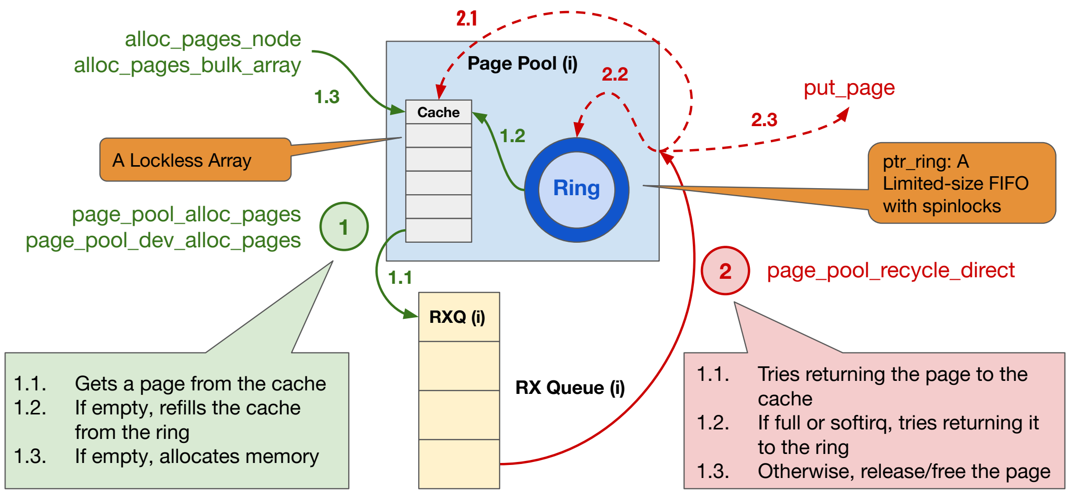

Figure 20. Page Pool overview.

Details of the steps in Figure 20:

(1) **Allocating a page.** When a driver asks for a page: (1.1) the page pool initially checks the lockless cache; (1.2) if empty, it tries to refill the cache from the pages recycled in the ring; (1.3) if not possible due to software interrupts (softirq) or unavailability, it allocates page(s) and refills the cache. Recent Linux kernels perform bulk allocation (*e.g.,* 64 pages at a time) to amortize the allocation cost.

(2) **Returning a page.** When a driver returns a page: (2.1) the page pool attempts to recycle the page into the cache; (2.2) if not possible, it tries returning it to the ring; (2.3) if unsuccessful, it releases/frees the page. Since the Page Pool API is optimized for eXpress Data Path (XDP) and AF_XDP socket (XSK), *i.e.,* an eBPF data path where each page is only used by one buffer, Steps 2.1 & 2.2 can only be performed if (i) a page has only a *single* reference (*i.e.,* `page_ref_count(page) == 1`) and (ii) a page is *not* allocated from the emergency pfmemalloc reserves. The former causes a page pool to continually allocate new pages for drivers operating in a non-XDP mode that splits each page into multiple fragments and uses page references for bookkeeping/recycling. Unfortunately, continuous page allocation disables the driver from re-using a fixed set of pages, causing low page locality. We refer to this as the *leaky page pool* problem.
