# Supplementary material for: Overcoming the IOTLB wall for multi-100-Gbps Linux-based networking
Source: PeerJ Comput Sci. 2023 May 16;9:e1385. doi: 10.7717/peerj-cs.1385 (PMC10280580; doi:10.7717/peerj-cs.1385)
Supplement: Supplemental Information 4 [file peerj-cs-09-1385-s004.pdf]

D CURRENT IMPLEMENTATION OF HPA

The current implementation of HPA extends the Page Pool API The kernel development community (2021) with a backup ring (`ptr_ring`) data structure to store a set of hugepage-backed 4-KiB pages; we chose the `ptr_ring` data structure due to its synchronization efficiencies.

We (*i*) allocate 2-MiB hugepages (via the buddy page allocator[15]), (*ii*) map them to IOMMU, (*iii*) keep the mappings in a doubly linked-list to be able to unmap the pages at the page pool's destruction, and (*iv*) store the sub-4-KiB pages in the backup ring. While it is possible to store the pre-allocated memory in the existing cache or ring data structures, we consciously decided not to do so to avoid affecting the existing machinery. In our approach, the backup ring is only used to refill the cache when the page pool needs to allocate memory (see step 1.3 in Figure 20). If the driver is *not* leaky, then the backup ring would never be empty (*i.e.,* the pre-allocated memory is sufficient). However, drivers with a *leaky page pool* will eventually deplete the backup ring. We noticed that our NIC could deplete a 1-GiB per-queue memory (*i.e.,* 512$\times$512 pages) after $\sim$100 seconds when operating at 200 Gbps. Unfortunately, constantly refilling the backup ring with pages could become costly, as it requires using spinlocks. Therefore, when the backup ring is known to be depleted, we switch to an alternative mode where we allocate a new 2-MiB hugepage and *directly* inject its 512$\times$4-KiB pages into the cache data structure. To do so, we increase the maximum size of the cache data structure to 1024 to potentially reduce the frequency of run-time allocations. Note that the page pool returns the *unrecyclable* pages (*i.e.,* individual 4-KiB pages within 2-MiB hugepage) back to the kernel and only saves their IOVA mappings. Therefore, run-time allocations do not over-allocate memory, but they may fragment memory over a long time and thus make the application more CPU-bound. However, as long as the application falls into the IOTLB-bound region, our solution would improve its performance.

[15] It is currently limited to allocating order-11 (8-MiB) pages.
